# Nuclear import of influenza A viral ribonucleoprotein complexes is mediated by two nuclear localization sequences on viral nucleoprotein

**DOI:** 10.1186/1743-422X-4-49

**Published:** 2007-06-04

**Authors:** Winco WH Wu, Ying-Hua B Sun, Nelly Panté

**Affiliations:** 1 Department of Zoology, University of British Columbia, 6270 University Boulevard, Vancouver, BC, V6T 1Z4, Canada

## Abstract

**Background:**

The influenza A virus replicates in the nucleus of its host cell. Thus, entry of the influenza genome into the cell nucleus is necessary for establishing infection. The genome of the influenza A virus consists of eight single-stranded, negative-sense RNA molecules, individually packed with several copies of the viral nucleoprotein (NP) into ribonucleoprotein particles (vRNPs). These vRNPs are large, rod-shaped complexes containing a core of NP, around which the RNA is helically wrapped. The vRNPs are the entities that enter the nucleus, and their nuclear import must be mediated by nuclear localization sequences (NLSs) exposed on the vRNPs. NP contains at least two putative NLSs, one at the N-terminus (NLS1) and one in the middle (NLS2) of the protein. These NP NLSs have been shown to mediate the nuclear import of recombinant NP molecules. However, it remains to be determined which NLS mediates the nuclear import of influenza vRNP complexes.

**Results:**

To directly track the nuclear import of the influenza A genome, we developed an experimental assay based on digitonin-permeabilized cells and fluorescently-labeled vRNPs isolated from the influenza A virus. We used this assay to determine the contribution of the two proposed NLSs on NP to the nuclear import of influenza vRNP complexes. Peptides that mimic each of the two NLSs on NP were used to compete with vRNPs for their nuclear import receptors. In addition, antibodies against the two NP NLSs were used to block the NLSs on the vRNP complexes, and thereby inhibit vRNP nuclear import. Both peptide competition and antibody inhibition of either sequence resulted in decreased nuclear accumulation of vRNPs. The two sequences act independently of each other, as inhibition of only one of the two NLSs still resulted in significant, though diminished, nuclear import of vRNPs. Furthermore, when both sequences were blocked, vRNP nuclear import was almost completely inhibited. Antibody inhibition studies further showed that NLS1 on NP is the main contributor to the nuclear import of vRNPs.

**Conclusion:**

Our results demonstrate that both NLS1 and NLS2 on NP can mediate the nuclear uptake of influenza A vRNPs.

## Background

As part of its replication cycle, the genome of the influenza A virus must enter the nucleus of its host cell. The influenza A virus genome consists of eight single-stranded, negative-sense viral RNA (vRNA) molecules of varying sizes that are individually packed and stabilized by multiple copies of nucleoprotein (NP; ~56 kDa) into viral ribonucleoprotein (vRNP) complexes. NP forms a core around which the vRNA is helically wrapped [1]. Approximately 24 nucleotides associate with each NP [2,3]. Thus, given that each vRNA is about 890–2,341 nucleotides long (reviewed in [4]), each influenza vRNP has 37–97 copies of NP. The crystal structure of oligomeric NP has recently been solved and revealed a possible RNA-binding groove made up of a large number of basic residues [5]. In addition to NP, each vRNP also contains a single copy of a trimeric RNA polymerase complex. In the virus, these vRNPs are enclosed by the viral envelope, and are organized into a distinct pattern with seven vRNPs in a circle surrounding one vRNP at the center [6]. During cell entry, the influenza virion containing these incoming vRNP complexes is internalized into an endosomal compartment by either clathrin- or caveolae-dependent mechanisms [7,8]. The acidic environment of the endosome then triggers the fusion of the viral envelope with the endosomal membrane to release the vRNPs into the cytoplasm. The vRNPs are then transported in the cytoplasm by diffusion [9], and gain access to the nuclear import machinery of the cell (nuclear pore complexes (NPCs) and soluble nuclear import receptors). After reaching the NPCs, the vRNPs are then imported into the nucleus in an energy-dependent manner [9-11]. The nuclear import of incoming vRNPs allows for subsequent genomic replication; nuclear transcription and cytoplasmic synthesis of new viral proteins; nuclear import of newly-synthesized NP and RNA polymerases; and nuclear assembly and export of newly-synthesized vRNP complexes (reviewed in [12-15]).

Two mechanisms for nuclear import exist [16,17]: Passive diffusion occurs for molecules less than 9 nm in diameter, or proteins smaller than 40 kDa. Facilitated translocation, on the other hand, can accommodate molecules up to 39 nm in diameter [18]. This mechanism is highly selective and requires the energy from GTP hydrolysis by the small GTPase Ran [19,20]. In addition, facilitated translocation requires a signal residing on the imported molecule (or cargo), and soluble cytoplasmic receptors that recognize the signal and carry the cargo through the NPC. A major breakthrough in the study of nuclear import has been the recent identification of several signals and transport receptors mediating different nuclear transport pathways. The first-identified and best-studied nuclear import signal is characterized by one (monopartite) or two (bipartite) short stretches of basic amino acids, called nuclear localization sequences (NLSs) [21-23], and is now referred to as the classical NLS or cNLS. The receptor for the cNLS consists of two proteins, importin α and importin β. The cNLS is recognized by importin α, which acts as an adapter between the cNLS-bearing protein and importin β, the subunit which interacts directly with the NPC [24,25]. In addition, many other NLSs have been now identified (reviewed in [26]), and they bind different transport receptors that belong to an increasing family of proteins related to the importins.

Since influenza vRNPs enter the nucleus through the NPC via facilitated translocation [10], one or more exposed NLSs on the vRNPs must be responsible for mediating their nuclear import. Although all four proteins of influenza vRNPs (NP and the three RNA polymerases) carry NLSs, NP is believed to be the protein responsible for the nuclear import of influenza vRNPs. This hypothesis is based on experimental data that have demonstrated that NP mediates the nuclear import of *in vitro*-assembled NP-RNA complexes [27,28]. NP contains at least two NLSs: one at the N-terminus of NP (residues 1–13; NLS1) [29,30], and one in the middle of NP (residues 198–216; NLS2) [31]. Both sequences function as NLSs when fused to cytoplasmic proteins [30,31]. However deletion of both NLS1 and NLS2 still results in nuclear accumulation of NP, suggesting the presence of a third NLS [32], whose precise location is yet to be mapped. NLS1 has been studied in some detail and it is known that it binds to importin α1 and α5 [30], two of the six human homologues of importin αs currently identified (α1, α3, α4, α5, α6, α7) (reviewed in [33,34]). NLS2 is less well characterized, and was originally proposed to be a classical bipartite NLS. However, the recently-solved crystal structure of NP suggests that the two clusters of basic amino acids in NLS2 are too close to be recognized by importin α as a bipartite NLS [5]. Because mutations of the second cluster of basic amino acids (positions 213, 214 and 216) of NLS2 are critical to the nuclear import of recombinant NP [31], it is possible that this region may act as a monopartite NLS. The specific importin α homologue that recognizes NLS2 remains to be identified. However, since full length NP binds to importin α1, α3, and α5 [35], any of these homologues may recognize NLS2.

The contribution of NLS1 and NLS2 to the nuclear import of NP is controversial because they have been shown to mediate the nuclear import of recombinant NP in some studies but not in others [27,29,31,32]. Because NP forms small oligomers in equilibrium with monomers [5,36], it is unclear whether the NLSs on NP function during the oligomeric or the monomeric state of NP, and whether the different tags fused to recombinant NP in those studies may have interfered with the NLSs, or with the oligomerization of NP and consequent exposure of the NLSs. It is also unknown from these studies whether newly-synthesized NP enters the nucleus as oligomers or as monomers.

While the contribution of the NLSs to the nuclear import of newly-synthesized NP is controversial, even less is known about the specific NLS(s) that target incoming influenza virus-derived vRNP complexes to the nucleus. It has recently been shown that the nuclear import of *in vitro*-assembled NP-RNA complexes was mediated by NLS1 on NP [27]. However these studies used recombinant NP with GFP fused to its C-terminus, which may have interfered with the assembly of NP because according to the crystal structure of NP, oligomerization requires a tail loop close to the C-terminus (residues 402–428) [5]. Thus these *in vitro*-assembled RNPs may differ structurally from influenza-assembled vRNP complexes within mammalian cells. More recently, a reverse genetics approach, essentially involving the co-transfection of recombinant RNA and NP under the control of non-influenza promoters, has also noted an important role of NLS1 in mediating influenza RNA nuclear import [37]. Compared to *in vitro*-assembled RNPs or recombinant RNA and NP, however, it is possible that naturally-occurring, influenza virus-derived vRNPs may not actually utilize NLS1, or may also require NLS2, on NP for proper nuclear import. In this study, we address these key questions by studying the nuclear import of vRNP complexes isolated from naturally-occurring influenza A virions.

## Results

### Purification and biotinylation of influenza vRNP complexes

**Figure 1:**
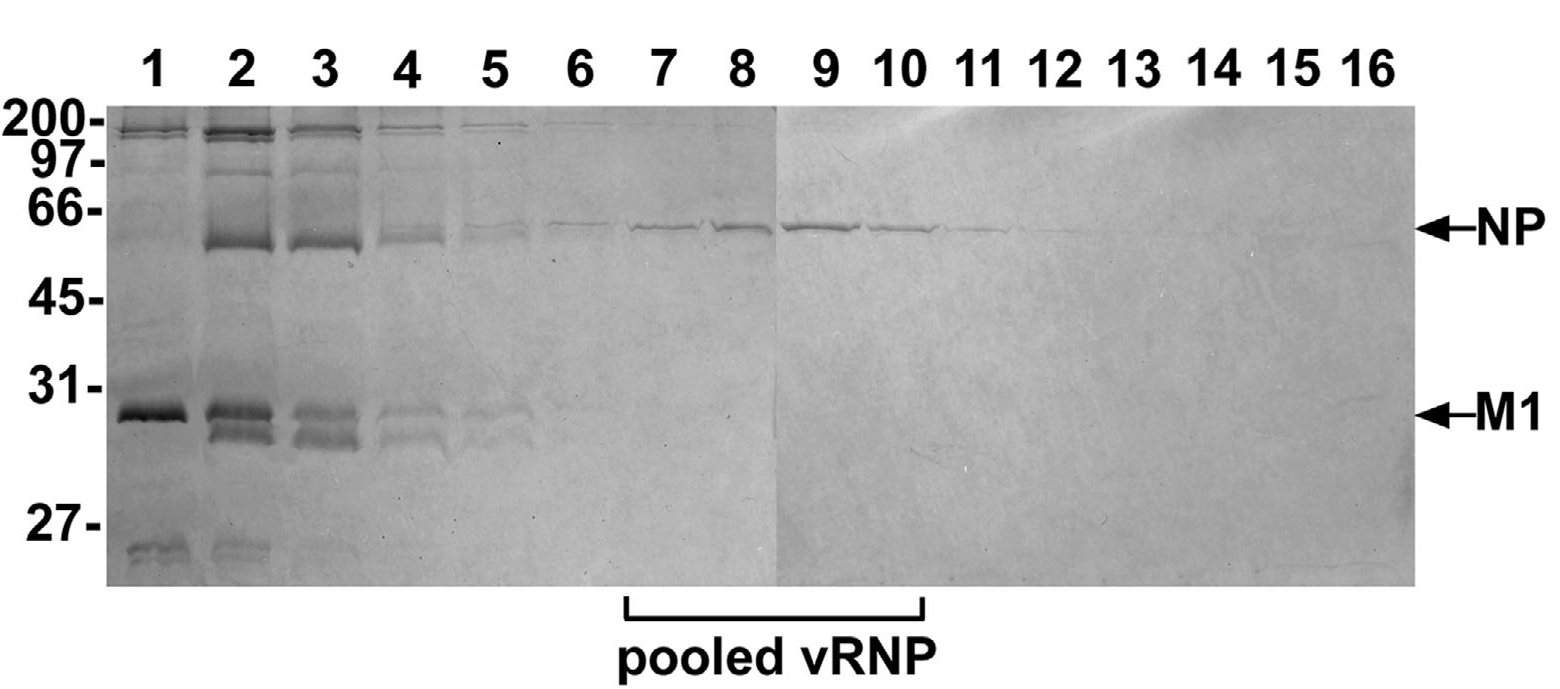
**Fractionation profile of the purification of vRNP complexes from influenza A by glycerol gradient centrifugation**. Fractions were collected from the top (lane 1) to the bottom (lane 16) of a glycerol gradient and analyzed via reducing SDS-PAGE containing 10% polyacrylamide. The gel was stained with Coomassie blue. The arrows indicate the mobility of the influenza NP and M1 proteins. The positions of the molecular weight standards (in kDa) are indicated to the left.

To study the nuclear import of the influenza genome it was important to first establish a method to purify and fluorescently label the influenza vRNPs in their native state. To purify the influenza vRNPs we used the well-established protocol of Kemler *et al. *[38], which consists of disrupting the influenza virions and then separating the released vRNPs from the other components of the virus by velocity sedimentation on a glycerol gradient. To mimic as closely as possible the conditions that influenza vRNPs undergo during cell infection after being internalized into endosomes, the vRNPs were purified under acidic conditions (pH 5.5). Fig. 1 represents a typical distribution profile of the proteins in the fractions. As previously shown [38], this procedure yields fractions containing vRNPs devoid of M1 and other influenza viral proteins, as judged by the presence of only the NP protein by Coomassie blue staining of the gel. Since M1 has been found to inhibit nuclear import of the vRNP complex [11,39], it was important to pool fractions containing NP but not M1 (as indicated in Fig. 1). These fractions, containing the vRNP complexes, were pooled, concentrated, and used for subsequent fluorescent labeling.

**Figure 2:**
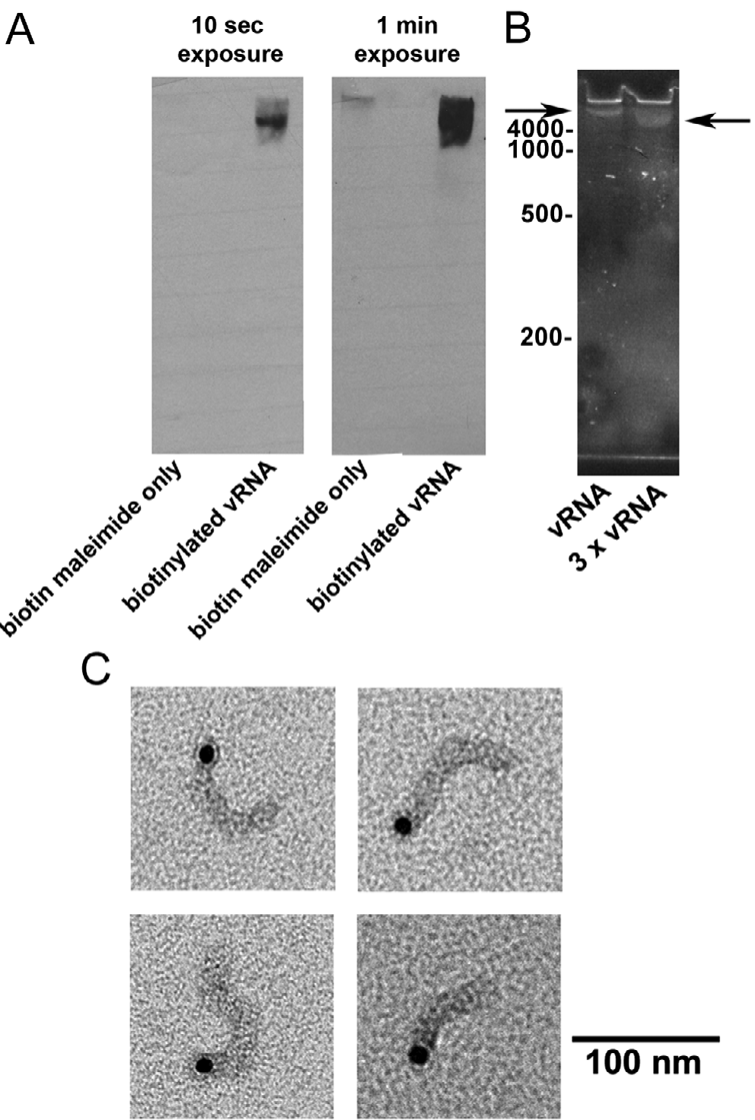
**Biotinylation of influenza vRNPs**. (A) Northern blot of biotinylated influenza vRNA in the vRNP complexes. The biotinylated vRNPs were subjected through urea gel electrophoresis, transferred onto nitrocellulose, and detected by blotting with streptavidin alkaline phosphatase. Shown are the results of the same blot exposed at two different times. As a control, biotin maleimide only was run on the gel. Urea gel electrophoresis of the same biotinylated influenza vRNA shown in A. (B) The vRNA within the vRNP complex was visualized directly by staining the gels with SYBR Safe. The arrows denote the positions of the influenza vRNA. Two different concentrations of the vRNA are shown. The positions of various sizes of RNA molecular standards (in bp) are shown on the left. (C) Electron microscopy visualization of specific binding of streptavidin-gold (10-nm diameter) to biotinylated influenza vRNPs. Gold particles exclusively associated with one end of the vRNPs.

To track the nuclear import of the influenza genome, the vRNA within the purified vRNP complex was biotinylated in order to attach a streptavidin fluorophore. For this purpose, we used the 5' End Tag Nucleic Acid Labeling System (Vector Laboratories), which covalently attached a reactive thiophosphate group to the 5' end of the vRNA within the vRNP. This allowed for the attachment of biotin-maleimide (a thiol-specific biotin reagent), and the subsequent tagging of fluorescein-streptavidin to yield fluorescein-labeled vRNPs. To verify that the vRNPs were properly biotinylated, their vRNAs were subjected to Northern blotting using streptavidin alkaline phosphatase. As shown in Fig. 2A, the vRNA was successfully biotinylated and ran near the top of the urea gel. Fig. 2B shows the same sample, but using the SYBR Safe dye to directly detect the vRNA on the urea gel. The stained vRNA (arrows), similar to the Northern blot, ran near the top of the gel. As the vRNA appeared to be larger than 4,000 bp and did not run as a smear or as distinct RNA bands, this indicates that the vRNA was still associated to the NP oligomer as part of the vRNP complex, and not dissociated from the protein components.

To confirm that biotin was added at one end of the vRNPs, the biotinylated vRNPs were incubated with streptavidin-gold, and the gold-tagged vRNP complexes were negatively stained and visualized by transmission electron microscopy. As illustrated in Fig. 2C, the vRNPs had the expected rod shape, and one end of the rod was labeled with an electron-dense gold particle.

### Fluorescein-labeled influenza vRNPs are competent for nuclear import

**Figure 3:**
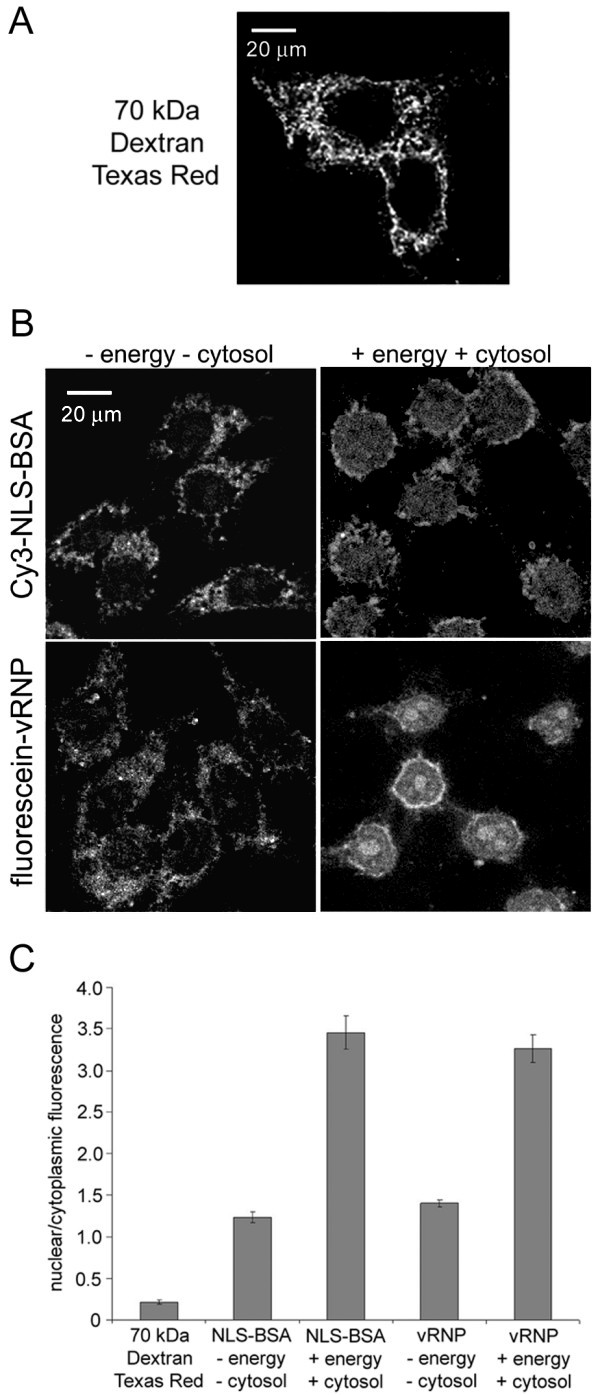
**Nuclear import of fluorescein-labeled influenza vRNPs**. Fluorescein-labeled influenza vRNPs are competent for nuclear import. Nuclear import assays were carried out in digitonin-treated HeLa cells, and cells were visualized by confocal microscopy. Representative images of three independent experiments are shown. (A) Control experiment with a 70 kDa dextran fluorescently-labeled with Texas Red to verify that the plasma membrane, but not the nuclear envelope, is permeabilized by digitonin. (B) Cy3-labeled BSA carrying a classical NLS (Cy3-NLS-BSA) and fluorescein-labeled influenza vRNP complexes (fluorescein-vRNP) were assayed in the digitonin permeabilized HeLa cells. Nuclear import assays were carried out in import buffer alone (- energy - cytosol) or in the presence of exogenous cytosol and an energy-regenerating system (+ energy + cytosol). (C) Bar diagram of the ratio of nuclear-to-cytoplasmic fluorescence for the experimental conditions shown in A and B. Each bar graph shows the mean value and standard error from 100–110 individual cells.

The nuclear import of fluorescein-labeled influenza vRNPs was studied in the well-established nuclear import assay involving digitonin-permeabilized cells [40]. Digitonin permeabilizes the plasma membrane of cells, but retains the integrity of the nuclear envelope; digitonin-permeabilized cells therefore have intact import-competent nuclei, but are depleted of cytosolic nuclear import receptors. To ensure that the nuclear import assay was working properly, a series of controls were performed. First, a fluorescently-labeled dextran with a size that exceeds the diffusion limit of the NPC was used to control for nuclear envelope integrity. As shown in Fig. 3A, the 70 kDa dextran labeled with Texas Red entered the plasma membrane but was almost totally excluded from the nucleus (indicated by the presence of fluorescence signal in the cytoplasm but not in the nucleus). This indicates that the plasma membrane was permeabilized, while the integrity of the nuclear envelope was maintained.

To ensure that the digitonin-permeabilized cells supported nuclear import, we used a positive control import substrate, the simian virus 40 large T antigen NLS cross-linked to BSA (NLS-BSA) and fluorescently labeled it with Cy3 (Cy3-NLS-BSA). As shown in Fig. 3B, Cy3-NLS-BSA efficiently accumulated in the nucleus of digitonin-permeabilized cells in the presence of exogenous cytosol and an energy-regenerating system. Quantitation of the fluorescence intensity in the nucleus and cytoplasm revealed that Cy3-NLS-BSA accumulated in the nucleus at levels 3.5-fold greater than in the cytoplasm (Fig. 3C). In contrast, in the absence of exogenous cytosol and an energy-regenerating system, Cy3-NLS-BSA was found mainly throughout the cytoplasm, and unable to accumulate effectively in the nucleus. Although quantitation of the fluorescence intensity indicated that a small amount of Cy3-NLS-BSA was in the nucleus in the absence of exogenous cytosol and an energy-regenerating system (Fig. 3C), this was probably due to residual cytosolic import factors and energy remaining in the permeabilized cells, which has been previously reported [24]. In agreement with this explanation, blocking the NPCs with the monoclonal antibody 414 prevented nuclear import (data not shown).

Next, we tested whether the fluorescein-labeled influenza vRNPs (fluorescein-vRNP) were competent for nuclear import. As illustrated in Fig. 3B, similar to Cy3-NLS-BSA, fluorescein-vRNP efficiently accumulated in the cell nucleus under permissive conditions (+cytosol + energy). As revealed from the quantitation of the fluorescence intensity (Fig. 3C), the level of nuclear accumulation of fluorescein-vRNP under permissive and non-permissive conditions was comparable to that of Cy3-NLS-BSA. Consistent with previous reports, the vRNP complexes were especially prone to accumulation at the nucleolus [37,41].

Taken together, these results demonstrate that the nuclear import assay with digitonin-permeabilized cells is functional, and that the fluorescein-labeled vRNPs are competent for nuclear import.

### Peptides mimicking NLS1 or NLS2 on NP inhibited the nuclear import of influenza vRNPs

**Figure 4:**
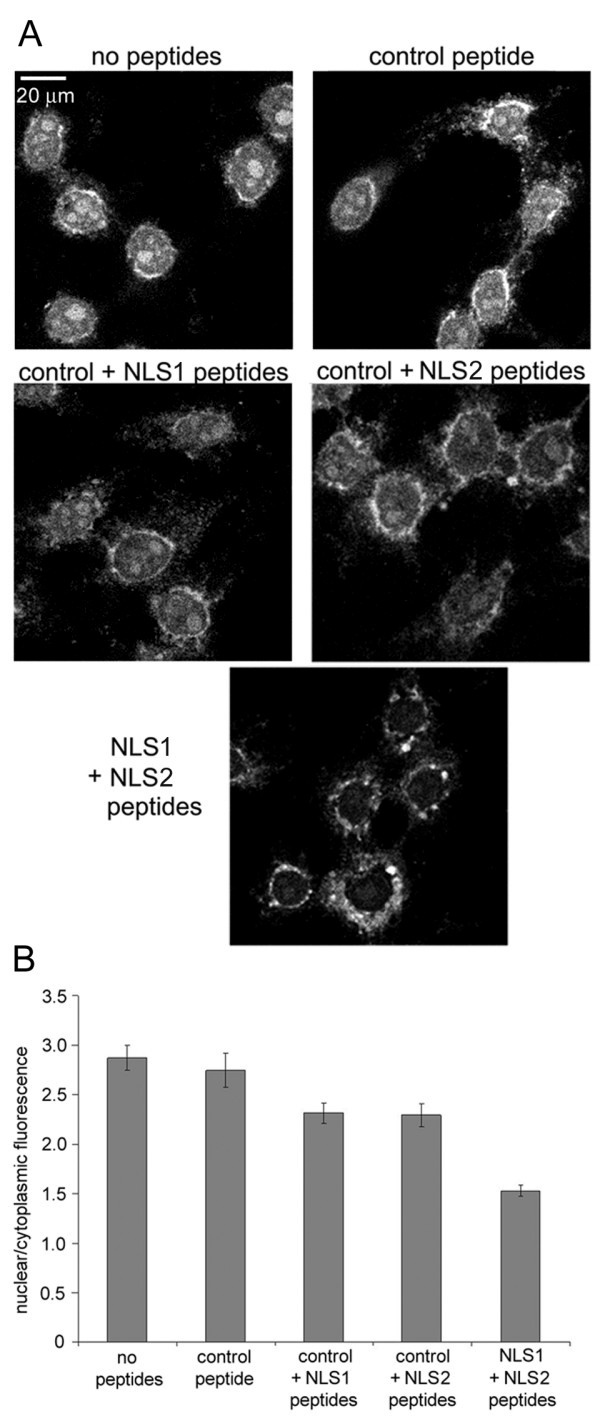
**Competition of influenza vRNP nuclear import with peptides against NP NLSs**. Peptides carrying the NLSs of influenza NP compete for nuclear import of influenza vRNPs. (A) Fluorescein-labeled influenza vRNPs were assayed in digitonin-permeabilized HeLa cells in the presence of cytosol, an energy-regenerating system, and the absence or presence of different peptides. Cells were visualized by confocal microscopy, and representative images of three independent experiments are shown. (B) Bar diagram of the ratio of nuclear-to-cytoplasmic fluorescence for the experimental conditions shown in A. Each bar graph shows the mean value and standard error from 100–110 individual cells.

To understand the contributions of the two putative NLSs on NP to the nuclear import of influenza vRNPs, peptides that mimic the NP NLS1 or NLS2 were used to compete with the vRNPs for their nuclear import receptors (the importin αs). Our hypothesis for these peptide competition experiments was that if a peptide served as an NLS that is responsible for the nuclear import of influenza vRNP, that peptide would bind to its nuclear import receptor. Thereby, the receptor would be unavailable to bind to and import the vRNPs into the nucleus. Figure 4A shows a representative peptide competition experiment performed with fluorescein-vRNPs in digitonin-permeabilized cells. As shown in Fig. 4A, a control peptide consisting of a mutated NLS [42] did not inhibit the nuclear import of fluorescein-vRNPs. Similarly, quantitation of the nuclear-to-cytoplasmic fluorescence in conditions with and without control peptide revealed that the nuclear accumulation of fluorescein-vRNP was similar in the two samples. In contrast, nuclear accumulation of fluorescein-vRNP was reduced in the presence of either the NLS1 peptide or the NLS2 peptide. Moreover, nuclear import of fluorescein-vRNP was further reduced when both NLS1 and NLS2 peptides were included.

The results displayed in Fig. 4 are for conditions in which the NLS1 peptide or NLS2 peptide was included in the presence of the control peptide added at a 1:1 ratio. (All conditions contained the same total molarity of peptides, so the control peptide was added to maintain constant peptide concentrations when comparing with the NLS1 + NLS2 double peptide competition condition.) Experiments were also performed with just the NLS1 peptide or NLS2 peptide in the absence of a control peptide, and a similar inhibition profile was obtained (results not shown). In either case, inhibition of either NLS1 or NLS2 alone did not completely inhibit the nuclear import of the fluorescein-vRNP. This indicates that NLS1 and NLS2 act independently of each other to promote nuclear import of the vRNPs since inhibition of only one NLS still resulted in a certain degree of vRNP nuclear accumulation. Furthermore, this inhibitory effect was additive because when a combination of NLS1 + NLS2 peptides were added, a significantly larger reduction in the nuclear import of the vRNPs occurred (Fig. 4).

### Antibodies against NLS1 or NLS2 of NP inhibited the nuclear import of influenza vRNPs

**Figure 5:**
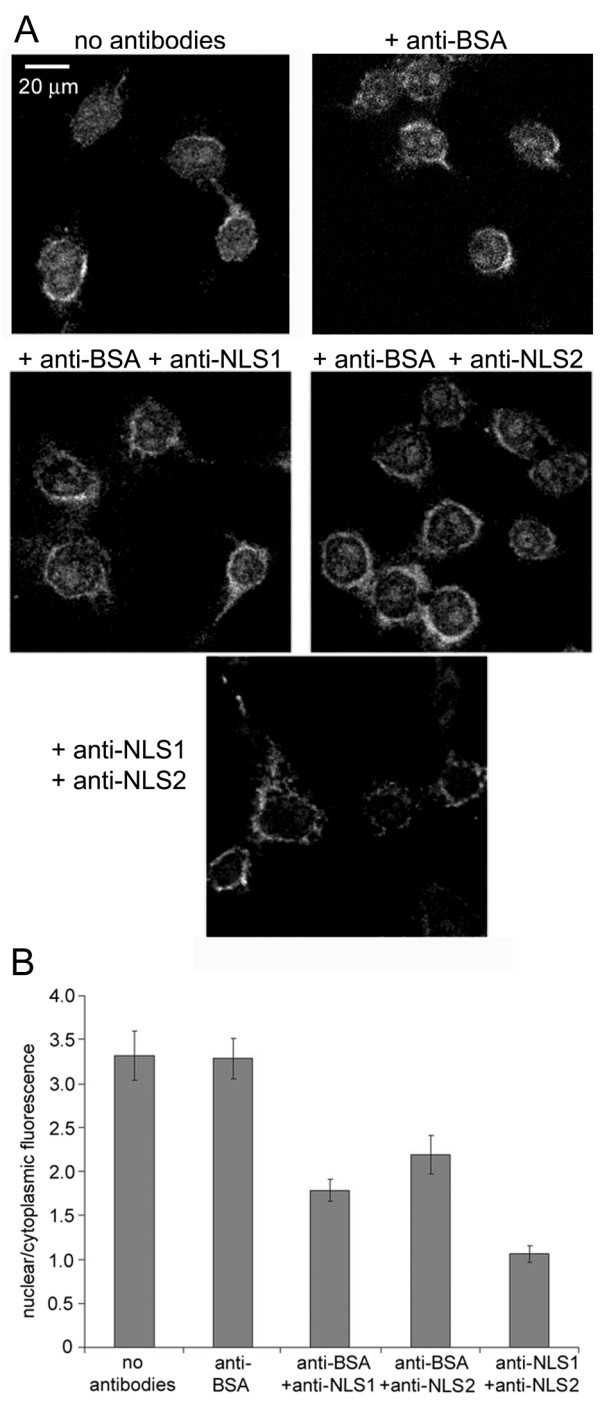
**Inhibition of influenza vRNP nuclear import with antibodies against NP NLSs**. Antibodies against the NLSs of influenza NP inhibit the nuclear import of influenza vRNPs. (A)Fluorescein-labeled influenza vRNPs were assayed in the digitonin-permeabilized HeLa cells in the presence of cytosol, an energy-regenerating system, and the absence or presence of different antibodies. Cells were visualized by confocal microscopy, and representative images of three independent experiments are shown. (B) Bar diagram of the ratio of nuclear-to-cytoplasmic fluorescence for the experimental conditions shown in A. Each bar graph shows the mean value and standard error from 100–110 individual cells.

To verify the results of the peptide competition experiments, a second approach consisting of antibody inhibition was used. For this purpose, antibodies against NLS1 or NLS2 of NP were applied in the nuclear import assay with digitonin-permeabilized HeLa cells using fluorescein-vRNP in the presence of exogenous cytosol and an energy-regenerating system. Our hypothesis was that these antibodies would bind to the vRNP complexes and prevent the association of importinα to the vRNPs, thereby inhibiting fluorescein-vRNP nuclear import. As a control antibody, we applied anti-BSA. As shown in Fig. 5, the anti-BSA control antibody did not affect the nuclear import of fluorescein-vRNP. However, in the presence of either the anti-NLS1 or anti-NLS2 antibodies, nuclear accumulation of fluorescein-vRNP was substantially decreased. Furthermore, similar to the peptide competition studies, antibody inhibition of both NLS1 and NLS2 resulted in an even greater decrease in fluorescein-vRNP nuclear import compared to inhibition with a single antibody (Fig. 5). Results shown in Fig. 5 are for experiments in which the control antibody was included with the anti-NLS1 antibody or anti-NLS2 antibody to maintain a constant total antibody concentration. However, similar results were obtained when only the anti-NLS1 antibody or anti-NLS2 antibody was added without the control antibody (results not shown).

The antibody inhibition results generally agree with those of the peptide competition experiments described above (Fig. 4). However, inhibiting the NLSs with antibodies seemed to be more effective in inhibiting the nuclear import of the fluorescein-vRNPs, than competing with these sequences. Furthermore, a difference was observed in the inhibition of NLS1 versus NLS2 with the antibodies, but not with the peptides. The discrepancy between these antibody results, which showed a difference between the two NLSs versus the peptide competition results, occurred probably because the antibodies directly inhibited nuclear import by blocking the NLSs on the vRNP, while the peptides indirectly inhibited nuclear import by competing for the vRNP nuclear import receptors. If so, the antibody inhibition results would indicate that while both NLS1 and NLS2 of NP may be involved in mediating the nuclear import of virally-derived vRNP complexes, the contribution of NLS1 is slightly greater.

## Discussion

Using influenza vRNPs purified under acidic conditions, and thus mimicking physiologically-relevant influenza infections as closely as possible, we have found that inhibition of either NLS1 or NLS2 on influenza NP significantly inhibited the nuclear import of influenza vRNP complexes. Therefore, both NLS1 and NLS2 on NP are involved in mediating the nuclear import of incoming vRNP complexes. These two sequences act independently of each other, as peptide competition with or antibody inhibition of only one of these sequences still resulted in a certain, though less pronounced, degree of nuclear import of vRNPs. Furthermore, when both NLS1 and NLS2 were competed with peptides or blocked by antibodies, the nuclear import of the vRNPs was even more drastically reduced.

Some differences, however, existed in the ability of the NLS peptides versus the anti-NLS antibodies to inhibit nuclear import of the vRNPs. These differences were likely due to the nature of the competition experiments, since peptide competition with the vRNPs for the cytosolic nuclear import receptors is less specific than direct inhibition of the vRNP NLSs with antibodies. The antibody inhibition experiments may hence provide a more accurate picture of the relative contributions of NLS1 and NLS2 to influenza vRNP nuclear import. From these results, it appears that peptides mimicking or antibodies against these conserved NLS regions on NP may be an effective means of interrupting a critical stage in the influenza A life cycle.

Interestingly, performing a sequence alignment (using Clustal W [43]) of NP from different influenza A strains, we can observe that each of NLS1 and NLS2 on NP are highly conserved among influenza A strains. However, NLS1 and NLS2 do not share much similarity with any region on NP of influenza B or C, as analyzed from a Clustal W alignment. This is in agreement with previous studies that have not been able to pinpoint the NLS in NP for influenza B [44], so it appears that influenza B and C may utilize NLSs that are different from those of influenza A. With respect to NLS1, the only residue conserved among influenza A, B, and C is a conserved arginine at position 8 of the influenza A NP, which agrees with previous studies indicating that that residue is one of the most important residues involved in mediating the nuclear import of recombinant NP [29,30]. For NLS2, residue 214 on influenza A NP is probably one of the most important residues as an arginine or lysine is found in that position in influenza A, B, and C.

The exact length of NLS2 also remains to be determined. Previous studies had implicated that NLS2 was a bipartite NLS of 19 amino acids long, spanning residues 198 to 216 [31]. However, recent structural data has questioned that this NLS functions as a bipartite classical NLS since the crystal structure of NP showed that the two clusters of basic residues of the bipartite NLS2 on NP were located too close together in space to be functional as a bipartite NLS [5]. Even though NLS2 may not be a bipartite NLS, the relevance of residues 213, 214, and 216 on NP in mediating the nuclear import of recombinant NP appears to be significant [31]. However, which other residues in NLS2 are important in mediating influenza nuclear import remains to be determined.

Our studies here with influenza vRNPs also confirm findings with recombinant NP that NLS1 is the stronger of the two NLSs [29,31,37]. From the crystal structure of NP [5], it is probably reasonable to assume that NLS1, being an N-terminal sequence near the edge of NP, may be more accessible to the binding of cytosolic nuclear import factors than NLS2. However, having both NLS1 and NLS2 as functional NLSs on influenza vRNPs could serve a vital purpose to the virus. If, in the event that the N-terminal NLS1 is inadvertently cleaved off by any proteases in its host cell, the vRNP may still have an extra NLS (NLS2) to mediate its nuclear import. Nonetheless, it appears that with so many copies of NP, it does seem to be a redundant function. However, it is not clear how many of these NLSs are exposed when NP oligomerizes and associates with the vRNA. Therefore, further studies at understanding the kinetics, cellular targets, conformational states, and role in viral replication of NLS1 and NLS2 will be required to bring further light to their roles in influenza cellular trafficking and replication.

Previous work by other groups have concentrated mainly on the nuclear import of recombinant NP [5,27-31,37]. These studies have provided a better understanding of the role of the various NLSs on NP in the nuclear import of newly-synthesized NP, which occurs after the initial nuclear import of the entire vRNP complex and the subsequent synthesis of new NP in the cytoplasm [12,14]. To study the nuclear import of influenza vRNPs, O'Neil *et al. *(1995) [27], and more recently Cros *et al. *(2005) [26], formed *in vitro*-assembled NP-RNA complexes by incubating recombinant NP with *in vitro*-synthesized influenza vRNA. To study the nuclear import of the influenza genome, however, vRNPs purified from influenza virions would likely be the preferred substrates over *in vitro*-formed vRNA-NP complexes. This is because the actual assembly of NP into an oligomeric structure, and the interactions of NP with the vRNA in actual influenza infections, would likely result in structural differences between *in vitro*-formed RNA-NP complexes versus virally-produced and assembled vRNPs. For example, certain NLSs may be exposed or hidden according to how NP actually interacts with itself in the oligomer and how NP interacts with the vRNA. In addition, NP molecules within actual influenza vRNPs that are produced in mammalian cells may have differences in their post-translational modifications compared to recombinant NP molecules produced in bacteria. Furthermore, any conformational changes in the structure of the vRNPs after influenza export from the cell, viral entry into a new cell, and during or after their exit from endosomes are not taken into account by *in vitro*-formed RNA-NP complexes. The methodological differences in the preparation of vRNPs may therefore explain the differences observed in studies using *in vitro*-formed RNP and our results reported here using naturally-occurring, influenza-derived vRNP complexes. For example, Cros *et al. *[27] found that disruption of NLS2 on NP has no effect on the nuclear accumulation of *in vitro*-formed vRNA-NP complexes, while we showed here that interfering with NLS2 on NP diminished the nuclear import capability of influenza-purified vRNPs (Figs. 4 and 5). Likewise, Ozawa *et al. *[37] found that NLS2, but not NLS1, deletion mutants of NP were unable to target effectively to nucleolar regions, while we showed here that nucleolar localization still occurred whether NLS1 or NLS2 on NP was inhibited.

Much work on understanding the nuclear import of the influenza genome still remains. For example, the nuclear accumulation sequence (NAS) within influenza-assembled vRNP complexes is still a mystery. The NAS on NP was originally found to mediate nuclear accumulation of NP in *Xenopus *oocytes [45], but more recently has been found to be a cytoplasmic retention signal in mammalian cells [30,31,46]. It would therefore be useful to understand in greater detail the role of the NAS and how its function relates to that of NLS1 and NLS2. In addition, the role of M1 in preventing nuclear import of vRNP is still unclear [11,39]. For example, M1 may be acting indirectly, where interaction of M1 with the vRNPs causes the vRNPs to change their structural conformation [47] and thus expose or hide certain NLSs. Alternatively, M1 may be binding directly to the NLSs of vRNPs to inhibit their nuclear import. The studies completed to date also raise the question as to under what conditions and in what conformational states of NP would NLS1 and NLS2 act to mediate the nuclear import of vRNPs. Further unraveling the answers to these questions may give us a more detailed understanding of how the various NLSs on the influenza vRNPs work together or independently to mediate influenza A nuclear import.

## Conclusion

In summary, we have showed in this study that inhibition of either NLS1 or NLS2 on NP from influenza-derived vRNP complexes significantly decreased the extent of nuclear localization of the influenza genome. Furthermore, inhibiting both NLSs resulted in an additive effect, causing an even greater decrease in vRNP nuclear accumulation. This indicates that both NLS1 and NLS2 on NP play a critical role in nuclear import by acting independently to mediate the nuclear import of incoming influenza A vRNP complexes. The importance of our findings in the design and development of novel influenza antiviral therapeutics is critical, as both NLSs will likely require to be inhibited to more completely abolish influenza nuclear import and thus viral replication.

## Methods

### Purification of influenza vRNP complexes

Virally-derived influenza vRNPs were purified according to Kemler *et al. *[38] with minor modifications: 1 ml of the H3N2 X-31 A/AICHI/68 strain of influenza A (Charles River Laboratories, Wilmington, MA) at 2 mg ml^-1 ^was washed in 30 mM Tris, pH 7.5, 20 mM MES, and 150 mM NaCl, and then centrifuged for 10 minutes, 4°C, at 109,000 × *g *using a TLA-120.2 rotor in an Optima Max-E centrifuge (Beckman Coulter, Fullerton, CA). The pellet was resuspended in 0.5 ml disruption buffer (100 mM MES, pH 5.5, 100 mM KCl, 5 mM MgCl_2_, 5% glycerol, 50 mM octylglucoside (Sigma, St. Louis, MO), 10 mg ml^-1 ^lysolecithin (Sigma), and 1.5 mM dithiothreitol (Sigma)). After shaking and vortexing at 31°C, this sample was loaded onto a glycerol gradient containing 1 ml 70% glycerol, 0.75 ml 50% glycerol, 0.375 ml 40% glycerol, and 1.8 ml 33% glycerol in buffer containing 50 mM MES, pH 5.5, and 150 mM NaCl. Centrifugation was performed for 3.75 hours at 4°C in an MLS-50 rotor at 217 000 × *g*. Fractions were analyzed on a reducing SDS gel containing 10% polyacrylamide. Gels were stained in 0.025% Coomassie brilliant blue G-250 (Sigma). Peak fractions containing the vRNPs were pooled, washed in Ultrapure DEPC-treated water (Invitrogen, Carlsbad, CA) by ultracentrifugation in a TLA-120.2 rotor at 157,000 × *g *for 4.5 hours at 4°C, and concentrated by resuspending the pellets in DEPC-treated water. The A_280 _of the concentrated vRNP was measured, and samples were aliquoted and frozen at -80°C. As NP is the major component present in the pooled fractions, an estimate of the molarity of NP was calculated using its extinction coefficient of 55,350 M^-1 ^cm^-1 ^(as determined via ProtParam [48]), to calculate the amount of peptides or antibodies required for the competition and inhibition studies.

### Biotinylation of influenza vRNP complexes

The 5' EndTag Nucleic Acid Labeling kit (Vector Laboratories, Burlingame, CA) was used to covalently attach a reactive thiophosphate group to the 5' end of the vRNA in the vRNP complex: The 5' phosphate from the vRNA was removed with alkaline phosphatase at 37°C for 30 minutes, followed by transferal of the thiophosphate from ATPγS to the vRNA 5' end with T4 polynucleotide kinase at 37°C for 30 minutes. Biotin-maleimide (Sigma), which binds to the sulfur on the thiophosphate, was then incubated with the thiophosphorylated vRNP complex at 65°C for 30 minutes.

### Urea gel electrophoresis and Northern blotting of biotinylated vRNA

Purified influenza vRNPs were diluted in loading dye containing TBE buffer (45 mM Tris, pH 8, 45 mM boric acid, 1 mM EDTA), 0.1% SDS, 0.08 mg ml^-1 ^yeast RNA (Roche Applied Science, Basel, Switzerland) to scavenge any RNases, glycerol, and the tracking dyes bromophenol blue and xylene cyanol. The sample was heated at 93°C for 3 minutes, and then applied to a urea polyacrylamide gel (8.3 M urea and 6% polyacrylamide in TBE buffer). The vRNA was visualized by staining for 30 minutes at room temperature with the SYBR Safe dye (Invitrogen). RNA molecular weight standards (Sigma) were used to determine the relative mobility of the vRNA.

For Northern blotting, the unstained gel was transferred onto nitrocellulose, and blotted with the UltraSNAP Detection Kit (Vector Laboratories). This kit contained alkaline phosphatase-streptavidin to allow for the visualization of biotinylated influenza vRNA.

### Negative staining and transmission electron microscopy

Biotinylated vRNPs were incubated with streptavidin-gold (10-nm diameter) (Ted Pella, Redding, CA) overnight at 4°C. The sample was then absorbed onto 2% parlodion/carbon-coated electron microscopy grids for 5 minutes, washed with buffer containing 20 mM Tris, pH 7.4, and 120 mM KCl, and negatively stained with 1% uranyl acetate. Grids were visualized in a Hitachi H7600 transmission electron microscope (Hitachi High Technologies America, Schaumburg, IL).

### Conjugation of nuclear import substrates with fluorophores

As a control, bovine serum albumin (BSA) was covalently attached to a peptide (CGGGPKKKRKVED) containing the NLS of the simian virus 40 large T antigen at a ratio of 5:1 of NLS:BSA (conjugated by Sigma Genosys). The NLS-BSA was then conjugated to the Cy3 fluorophore (Amersham Biosciences, Piscataway, NJ), via incubation with 0.1 M sodium bicarbonate, pH 9.3, for 1 hour at room temperature. Biotinylated vRNP was conjugated to streptavidin-fluorescein (Vector laboratories) by incubation with streptavidin-fluorescein for one hour on ice.

### Nuclear import assay in digitonin-permeabilized HeLa cells

Adherent HeLa cells (American Type Culture Collection) were grown in a 37°C incubator containing 5% CO_2 _in Dulbecco's modified Eagle medium (HyClone, Logan, UT) supplemented with 9% fetal bovine serum (Sigma), penicillin-streptomycin (Sigma), 1 mM sodium pyruvate (Cellgro, Herndon, VA), and 2 mM L-glutamine (Cellgro). The nuclear import assay is based on that of Adam *et al. *[40]. Briefly, the HeLa cells were seeded onto glass cover slips such that they were 60–70% confluent the next day. The HeLa cells were then rinsed with import buffer (20 mM HEPES, pH 7.4, 110 mM potassium acetate, 1 mM EGTA, 5 mM sodium acetate, 2 mM magnesium acetate, and 2 mM dithiothreitol), and permeabilized with 20 μg ml^-1 ^digitonin in import buffer at room temperature for 5 minutes. After further washes with import buffer, the cover slips containing digitonin-permeabilized cells were inverted and incubated with the import mixture containing Cy3-labeled cNLS-BSA or fluorescein-labeled vRNP for 45 minutes at 37°C in the presence or absence of exogenous 20% cytosol (rabbit reticulocyte lysate, Promega (Madison, WI)) and an energy-regenerating system (0.4 mM ATP, 0.45 mM GTP, 4.5 mM phosphocreatine and 18 U ml^-1 ^phosphocreatine kinase (all from Sigma)). To prevent nonspecific binding, 1.6 mg ml^-1 ^BSA (Sigma) was added. Protease inhibitors (chymostatin, leupeptin, antipain, and pepstatin, all from Sigma) at 10 μg ml^-1 ^were also included. The cells were then washed, fixed with 4% paraformaldehyde in import buffer, and mounted with the Prolong Gold antifade reagent containing DAPI (Invitrogen). As a control, 70 kDa dextran Texas Red (Invitrogen) was applied to ensure that permeabilization of the plasma membrane, but not the nuclear membrane, had occurred [49]. In some experiments 100 μg/ml of the monoclonal antibody MAb414 (Covance), which recognizes proteins of the NPC and block nuclear import, was used.

### Peptide competition studies

Peptides bearing the sequences of NLS1 (^1^MASQGTKRSYEQM^13^) and NLS2 (^198^KRGINDRNFWRGENGRKTR^216^) on influenza A NP were synthesized by Pacific Immunology (San Diego, CA). As a control peptide, a mutated version of a cNLS-bearing peptide (CYTPPKTKRKV), which contains a threonine (underlined) at position 7 instead of a lysine and thereby does not support nuclear import [42], was used (synthesized by Sigma Genosys). For the peptide competition experiments, a 500-fold molar excess of the NLS1 or NLS2 peptides (500:1 of peptides: NP) was used in the nuclear import assay. For these experiments, the peptides were pre-incubated with exogenous cytosol for one hour at 4°C prior to addition of fluorescein-vRNP and initiation of the import assay. To maintain constant peptide concentrations, a 1000-fold molar excess of combined peptides was used for each sample. The control peptide was therefore used where only a single NLS1 or NLS2 peptide was added. (For example, a 1000-fold molar excess of control peptide only was used for the control sample, and a 500-fold excess of control peptide + 500-fold excess of NLS1 peptide was used for the NLS1 peptide sample). Experiments were also performed with an excess of just the NLS1 or NLS2 peptides without containing control peptide.

### Antibody inhibition studies

Polyclonal antibodies to the above peptides mimicking NLS1 or NLS2 on NP were produced and affinity purified by Pacific Immunology. The specificity of these antibodies was checked by both dot blots and Western blots. Both antibodies specifically reacted with their correspondent peptide and with purified vRNPs.

For the nuclear import studies, each antibody was used at an eight-fold molar excess (8:1 of antibodies:NP) in the nuclear import assay. Anti-BSA (Sigma) was used as a control antibody. Antibodies were initially incubated with the fluorescein-vRNP for one hour at 4°C prior to initiation of the import assay. To maintain constant antibody concentrations, a 16-fold molar excess of combined antibodies was used for each sample. The control antibody was therefore used where only a single antibody was added. Experiments were also performed with just the anti-NLS1 or the anti-NLS2 antibodies without containing control antibody.

### Fluorescence microscopy

Conventional fluorescence microscopy was performed on a Zeiss Axioplan 2 (Carl Zeiss, Oberkochen, Germany). Confocal laser scanning microscopy was performed on a Zeiss Pascal LSM 5 (Carl Zeiss).

### Quantification of nuclear import

To quantify the nuclear import of the fluorescently-labeled cargo, the ratio of the nuclear-to-cytoplasmic fluorescence signal was determined. For this purpose, the intensity of the nuclear and cytoplasmic fluorescence was measured using ImageJ (National Institutes of Health, Bethesda, MD) according to Schedlich et al. [49]. Cells imaged under conventional fluorescence microscopy were used for quantitation because it more accurately reflected the total amount of fluorescence in the nucleus and the cytoplasm, as opposed to confocal microscopy, which only imaged the cells at one plane. To quantify the nuclear import, the background was first subtracted with ImageJ. Next the mean intensity of a defined area (20 pixels by 20 pixels) in the nucleus was measured and divided by the mean intensity of the same amount of area in the cytoplasm from the same cell. The area of the nucleus was chosen as close to the centre of the nucleus as possible, usually covering about 80% of the nucleus stained. The cytoplasmic area was chosen to be representative of the fluorescence intensity of the entire cytoplasm of the cell, usually covering about 40% of the total cytoplasm. The staining of the nuclear envelope was not included in the quantification. As photographs were imaged at intensities below saturation, there should not have been any problems with oversaturation of a certain area of the cell. Between 100–110 cells were quantified for each condition.

## Competing interests

The author(s) declare that they have no competing interests.

## Authors' contributions

WWHW carried out the experiments and drafted the manuscript. YHBS performed the preliminary studies to work out certain experimental protocols, and commented on the manuscript. NP conceived the study and experimental design, coordinated the study, and helped to draft the manuscript. All authors read and approved the final manuscript.
